# Bandage Lenses in the Postoperative Care for Cataract Surgery Patients: A Substitute for Eye Patch?

**DOI:** 10.1155/2018/1493967

**Published:** 2018-05-15

**Authors:** Hang Song, Yingyu Li, Yan Zhang, Danna Shi, Xuemin Li

**Affiliations:** ^1^Department of Ophthalmology, Peking University Third Hospital, Beijing, China; ^2^Sino Japanese Union Hospital of Jilin University, Changchun, Jilin, China

## Abstract

**Purpose:**

To explore whether bandage lenses could be a safe and effective substitute for eye patch in the postoperative care for cataract surgery patients in terms of infection prevention, ocular impacts, and patient satisfaction.

**Methods:**

Patients who underwent cataract surgery were randomly divided into the eye patch group (Group A) and the bandage lens group (Group B). Bacterial culture samples were collected perioperatively from different sites. Evaluations of anterior segment condition and patient satisfaction were conducted on the first day of postoperative follow-up.

**Results:**

The positive rate of bacterial cultures in Group A was higher than that in Group B, but the difference was not statistically significant. Group B had significantly longer tear breakup time, higher tear meniscus height, and slightly better patient satisfaction than Group A.

**Conclusion:**

Bandage lenses can be used as a safe and effective substitute for eye patch in the postoperative care for cataract surgery patients. The Clinical Study registration number is ChiCTR-IOC-17012167.

## 1. Introduction

Endophthalmitis is one of the most severe complications after cataract surgery, which often leads to visual deprivation. There are several methods to reduce the risk of postoperative infection, such as using perioperative antibiotic eye drops [1], administering povidone-iodine 5% solution in the conjunctival sac prior to surgery [2, 3], using intracameral antibiotics [4], and prophylactic subconjunctival antibiotic injection at the conclusion of cataract surgery [3]. Wearing an eye patch for at least four hours after surgery was also considered as an effective measure to prevent bacterial contamination and protect the eyes from mechanical injury [1]. But the limitations of eye patch coverage have also become manifested [5, 6]. Research has shown that an eye patch might constrain lid closure, delay postoperative healing, increase discomfort, and is inapplicable to patients with monocular vision [6]. Given these limitations, attempts to remove postsurgical eye coverage have been made, which allow patients to gain the so-called ‘‘instant vision” [7]. A major difficulty, however, is that patients without any coverage would be subject to significantly higher discomfort and pain scores as well as significantly worse tear breakup time [8], and administering artificial tear eye drops could only partially improve the outcomes [7].

In this paper, we propose to use therapeutic contact lens to improve the current approach to “instant vision” by overcoming its side effects. Therapeutic contact lens, also known as “bandage lens,” is designed to protect and facilitate the healing of a sick eye. Serving as a blanket for the cornea, bandage lens retains its moisture, promotes epithelialization [9], blocks small wound leaks, relieves suture irritation [10], and smoothens wound margin irregularities. A modified bandage lens can also act as a drug delivery system [11, 12]. It is widely used in chronic epithelial defects, corneal ulcer, chemical burns, and post cornea-related surgery. Our study aims at evaluating the efficacy of the eye patch and bandage lenses in preventing eye infections after cataract surgery and investigating their ocular impacts and patient satisfaction level.

## 2. Methods

### 2.1. Study Design

This is a prospective, randomized, and controlled experimental study. Altogether, 52 subjects (52 eyes) were recruited at the Department of Ophthalmology, Peking University Third Hospital, from September 20th to November 1st, 2017. Informed consent was obtained from all participants. The study was approved by the Institutional Review Board of Peking University Third Hospital, and all the examinations were conducted in accordance with the tenets of the Declaration of Helsinki.

All the recruited patients had age-related cataract and were recommended for phacoemulsification and intraocular lens implantation surgery after an initial assessment. They were randomly assigned to either Group A or Group B. All patients underwent the same procedure of preoperative preparation. Three days before surgery, levofloxacin eye drops were administered four times a day. One hour before surgery, a nurse sequentially conducted conjunctiva washing with 50 ml of saline solution containing tobramycin at a concentration of 16 mg/ml, lacrimal passage irrigation with 3 ml of saline solution, and mydriatic treatment for six times for each patient. Phacoemulsification and intraocular lens implantation were performed by the same ophthalmologist right after an instillation of 5% povidone-iodine into the operated eye.

After surgery, patients in Group A received subconjunctival injection of tobramycin dexamethasone, started wearing eye patches on the same day, and began using anti-inflammatory eye drops the next day. Patients in Group B were instructed to wear therapeutic silicone hydrogel contact lenses (Bausch & Lomb Pure Vision, balafilcon A, New York, USA) on the day of surgery and started using anti-inflammatory eye drops since the day of surgery. The varieties of postoperative anti-inflammatory eye drops included levofloxacin, prednisolone, and pranoprofen, which were administered for one month at a frequency of four times per day in the first week, three times per day in the second week, twice per day in the third week, and once per day in the fourth week.

### 2.2. Inclusion and Exclusion Criteria

#### 2.2.1. Inclusion Criteria

The following are the various inclusion criteria:

1. patients who were diagnosed with age-related cataract
2. patients who were willing to receive phacoemulsification and intraocular lens implantation to improve their vision quality

#### 2.2.2. Exclusion Criteria

The following are the various exclusion criteria:

1. patients who have received eye operation within three months
2. patients who have worn contact lens within two weeks
3. patients with recurrent inflammation or eye traumas
4. patients with nasolacrimal duct obstruction
5. patients with prior use of systemic antibiotics and/or steroids in the past week
6. patients with severe ocular surface disorders

### 2.3. Data Collection

Five bacterial culture samples were collected with a cotton swab, respectively, from conjunctiva sac swabbing before conjunctiva washing and incision site and eyelid swabbing at the end of surgery as well as on the first day after surgery. For patients who wore bandage lenses, the soft contact lenses were removed with sterile forceps without any anesthetic drops, from which an extra culture sample was obtained in addition to the abovementioned five bacterial culture samples.

Follow-up was carried out on the first day after surgery by the same doctor. After bacterial culture samples were collected, a thorough eye examination of the anterior segment was performed. Tear film breakup time (TBUT) was employed to assess the stability of tear film and was measured three times in each case, from which an average value was calculated and adopted. Tear meniscus height was recorded and graded as 1 (0.1 mm to <0.2 mm), 2 (0.2 mm to <0.4 mm), or 3 (≥0.4 mm). The results of subconjunctival hemorrhage, conjunctival congestion, corneal swelling, whole corneal staining (incision site excluded), keratic precipitates, and anterior chamber flare were all evaluated with a dichotomous scale and recorded as absent or present. The subjects' feeling of pain, initial foreign body sensation, the foreign body sensation after eye patch or bandage lens removal, tearing, and photophobia were also measured with a dichotomous scale.

### 2.4. Bacterial Culture and Identification

Both swabbing samples and contact lens samples were smeared onto two types of media, respectively: one contained blood, and the other chocolate agar. The blood culture media plates were incubated to identify aerobic and microaerophilic bacteria. The chocolate agar plates were incubated in an anaerobic bag to isolate anaerobic bacteria. If any bacterium was found to grow within one week, the bacterium colony would be isolated for species identification using an automatic microbiology analysis system (Biomerieux VITEK 2 Compact).

### 2.5. Statistical Analysis

SPSS l3.0 (SPSS, Inc., Chicago, Illinois, USA) was employed for the statistical analysis of ocular surface conditions. Independent *T*-test analysis was performed to compare TBUT between the two groups. The chi-square test was used to analyze the dichotomous variables. Mann–Whitney *U* test was employed to analyze tear meniscus height. The level of statistical significance in this study was set at 0.05.

## 3. Results

### 3.1. Safety Evaluation Based on Bacterial Culture

The positive rate of bacterial cultures in different swabs is shown in Table 1. Altogether, there were four subjects in Group A and two in Group B whose cultures had positive results, and the distribution of positive cultures among these individual subjects is shown in Table 2.

### 3.2. The Evaluation of Anterior Eye Segment the Day after Surgery

On the first day after surgery, signs of the anterior segment of the operated eye were evaluated by the same doctor. Subconjunctival hemorrhage was observed at a significantly higher incidence in Group A, while conjunctival congestion, corneal swelling, whole corneal staining (incision site excluded), keratic precipitates, and anterior chamber flare showed no statistically significant difference between the two groups (Table 3). The TBUT test indicated that the tear film among patients in Group B was more stable than that in Group A (Table 4). The tear meniscus height in Group B was also significantly higher than that in Group A (*P*=0.015, data not shown).

### 3.3. Patient Satisfaction

Patients in neither group reported feelings of pain. Foreign body sensation was less complained in Group B than in Group A, but the variance was not statistically significant. However, foreign body sensation after coverage (eye patch or bandage lens) removal was more complained in Group B than in Group A. Complaint of tearing was nearly the same in both groups. As only the patients in Group B could see objects as soon as they finished operation, photophobia was only assessed in Group B, and two patients out of the 27 complained of it (Table 5).

## 4. Discussion

Covering the eye with an eye patch is a regular postoperative therapy for cataract surgery patients. However, the presence of secretions adhering to the eyelid is quite common when ophthalmologists take off the eye patch on the first day of postoperative follow-up. The secretions might increase the risk of infection and cause discomfort. If antibiotic ointment is administered at the conclusion of surgery, the discomfort would be more severe and the eye patch might even cause toxic anterior segment syndrome [13]. In addition, wearing an eye patch causes much inconvenience for patients with monovision, especially in outpatient surgery, after which the patient leaves the hospital immediately. Thus, it is important to explore proper substitutes for the eye patch, and in this study, we evaluated the plausibility of bandage lenses.

We developed bacterial cultures obtained from different sites and at various time points perioperatively. There was no positive culture on the first day after surgery, and the bandage lens cultures were all negative. Even though colonies of *Staphylococcus epidermidis* and *Staphylococcus aureus* were observed in the culture samples of the incision site at the end of surgery, the use of antibiotic eye drops on the day of surgery could kill the bacteria and therefore prevent infection. However, in Group A, positive cultures of *Staphylococcus epidermidis* were observed on the first day after surgery, even though none was found among those patients at the end of surgery. This might be accounted for by the bacterial contamination from the secretion of the meibomian glands [14] or conjunctiva, as an eye patch constrained the eyelid movement and blocked the process of self-scavenging. Since the etiologic agents of endophthalmitis following cataract surgery are genetically associated with bacterial flora in the conjunctiva, eyelid, and periorbital [15], immobilizing the eyelid and covering the eye may not be a good postoperative therapy to prevent infection. Although the bacterial cultures showed no statistically significant differences between these two groups, it did show a tendency that bandage lenses performed better or at least no worse than eye patch in preventing postoperative infection.

The anterior segment evaluation on the first day after surgery showed that bandage lenses played a positive role in tear film protection. Comparison of parameters like tear film breakup time and tear meniscus height implied that the tear film among patients wearing bandage lenses was more stable than those using an eye patch. Previous research has also indicated bandage lens could be a viable treatment option for the management of ocular conditions associated with dry eye diseases [16, 17]. Given that tear film is an important factor for cornea epithelial protection [18], bandage lens has its advantage over an eye patch in postsurgical cornea recovery.

Subconjunctival hemorrhage was observed at a significantly higher incidence in the eye patch group. This result could be attributed to the subconjunctival injection of tobramycin, as the operation itself might impair the microvessels and cause subconjunctival hemorrhage. In contrast, with bandage lenses, patients could use the antibiotic eye drops on the same day of surgery, thus sparing subconjunctival injection as well as the pain and subconjunctival hemorrhage it might cause.

Conjunctival congestion, corneal swelling, whole corneal staining (incision site excluded), keratic precipitates, and anterior chamber flare all showed no significant difference between the two groups. As we did these evaluations on the first day after surgery, there was not enough time for bandage lenses to produce a significantly different effect. Whether bandage lenses could improve these conditions needs further investigation.

Regarding the satisfaction levels, patients in neither group complained of feelings of pain. The results might be different if a same evaluation was conducted the second day after surgery, since the eye patch during the first day after surgery could constrain the eyelid movement and therefore prevent the massage of the incision site where epithelial cells are usually defective. With the eye covered, this pain could be neglected. Bandage lenses protect the cornea not only from potential exterior sources of injury but also from the impacts from the patient's own eyelids. The shearing effect created by the lids during the blink can inhibit reepithelialization and cause pain. Using bandage lens facilitates corneal healing in a pain-free environment.

Foreign body sensation was less complained of among patients wearing bandage lenses, though without statistical significance. However, foreign body sensation after bandage lens removal was more complained of than after eye patch removal. This, on the contrary, indicates that patients were more willing to wear bandage lenses than to wear nothing or eye patch. Only two patients out of the 27 in bandage lens group complained of photophobia, which indicated that the side effect of photophobia was at a low incidence for instant vision with bandage lenses. Besides, photophobia, which was usually reported after one week of postsurgical follow-up, might be attributed to the maladjustment to the increasing light that comes to the retina after the removal of cataract. Thus, photophobia might not be the side effect of bandage lens, but rather a common phenomenon after cataract surgery.

In this study, we removed the bandage lenses on the first day after surgery to carry out bacterial culture. In nonexperimental clinical scenarios, bandage lenses could be used for longer period with improved protection for the cornea. Research has already been done with longer postoperative use of bandage lenses and showed long-term improvement in the ocular surface conditions (unpublished data).

## 5. Conclusion

Using bandage lens is safe and effective in protecting ocular surface and stabilizing tear film after cataract surgery and has the advantage of maintaining the “instant vision” of the operated eye, which makes it a promising substitute for eye patch coverage in the postoperative care for cataract surgery patients, especially those with monovision.

## Figures and Tables

**Table 1:** Positive rate of bacterial cultures in different swabs.

Site and time point of swabs	Positive in Group A (*N*=25) *n* (%)	Positive in Group B (*N*=27) *n* (%)	*P* value^※^
Conjunctiva sac before surgery	0 (0)	0 (0)	1.000
Incision site at the end of surgery	1 (4)	0 (0)	0.481
Eyelid margin at the end of surgery	2 (8)	2 (7.4)	1.000
Incision site the first day after surgery	2 (8)	0 (0)	0.226
Eyelid margin the first day after surgery	2 (8)	0 (0)	0.226
Bandage lens culture	N/A	0 (0)	

^※^The result of the Fisher exact test was adopted.

**Table 2:** The distribution of positive cultures among individual subjects.

Patients with positive results	Conjunctiva sac before surgery	Incision site at the end of surgery	Eyelid margin at the end of surgery	Incision site the first day after surgery	Eyelid margin the first day after surgery	Bandage lens culture
Number 1 in Group A	(−)	*Staphylococcus epidermidis*	*Staphylococcus epidermidis*	(−)	*Staphylococcus epidermidis*	(−)
Number 2 in Group A	(−)	*Staphylococcus epidermidis*	(−)	(−)	(−)	(−)
Number 3 in Group A	(−)	(−)	*Staphylococcus epidermidis*	*Staphylococcus epidermidis*	(−)	(−)
Number 4 in Group A	(−)	(−)	*Staphylococcus epidermidis*	(−)	(−)	(−)
Number 1 in Group B	(−)	*Staphylococcus epidermidis*	(−)	(−)	(−)	(−)
Number 2 in Group B	(−)	*Staphylococcus aureus*	(−)	(−)	(−)	(−)

**Table 3:** Signs of the anterior segment evaluation.

Signs	Present in Group A (*N*=25) *n* (%)	Present in Group B (*N*=27) *n* (%)	*P* value^※^
Subconjunctival hemorrhage	4 (16)	0 (0)	0.047
Conjunctival congestion	11 (44)	11 (40.7)	1.000
Corneal swelling	15 (60)	16 (59.6)	1.000
Whole corneal staining (incision site excluded)	1 (4)	6 (22.2)	0.101
Keratic precipitates	13 (52)	18 (66.7)	0.397
Anterior chamber flare	16 (64)	23 (85.2)	0.112

^※^The result of the Fisher exact test was adopted.

**Table 4:** TBUT at the first day after surgery.

TBUT (s)	Mean	SD	*N*	*P* value
Group A	4.20	2.517	25	0.015
Group B	6.48	3.867	27	

**Table 5:** Subjective feelings on the first day after surgery.

Signs	Present in Group A (*N*=25) *n* (%)	Present in Group B (*N*=27) *n* (%)	*P* value^※^
Pain	0 (0%)	0 (0%)	1.000
Foreign body sensation	15 (60%)	10 (37.0%)	0.164
Foreign body sensation after coverage removal	0 (0%)	4 (14.8%)	0.112
Tearing	2 (8%)	3 (11.1%)	1.000
Photophobia	N/A	2 (7.41%)	

^※^The result of Fisher exact test was adopted.

## Data Availability

The original data used to support the findings of this study are available from the corresponding author upon request.
